# HMB-Net: Synergizing Latent Sequential Dynamics and Local Sequence Motifs for Robust RNA Methylation Prediction

**DOI:** 10.3390/biology15212028

**Published:** 2026-11-07

**Authors:** Fang Fang, Dong Xu, Rao Zeng

**Affiliations:** 1 School of Information Engineering, Hainan Vocational University of Science and Technology, Haikou 571126, China; fangf.0110@gmail.com; 2 School of Artificial Intelligence, Hainan Normal University, Haikou 571158, China; 3 Key Laboratory of Data Science and Smart Education, Ministry of Education, Hainan Normal University, Haikou 571158, China

**Keywords:** epitranscriptomics, RNA methylation, deep learning, Hierarchical learning, State Space Models, cross-species analysis

## Abstract

Accurate site-level identification of RNA post-transcriptional modifications is essential for deciphering epitranscriptomic regulation. However, existing deep learning predictors typically rely on either convolutional filters with limited receptive fields or self-attention mechanisms with quadratic complexity, while parallel hybrid networks often struggle to coordinate global sequence context with local motif sensitivity. Here, we present HMB-Net, an interpretable hybrid framework that couples a Selective State Space Model (Mamba) with a deep Bidirectional Long Short-Term Memory (Bi-LSTM) network in a sequential “Latent-to-Motif” hierarchy. Rather than extracting features in parallel, HMB-Net first employs a Mamba encoder to capture long-range sequence dependencies in linear time, which then guides a multi-layer Bi-LSTM to resolve fine-grained motif boundaries, followed by a bidirectional cross-attention module to integrate multi-scale representations. Comprehensive benchmarking across 20 mammalian datasets spanning *N*^6^-methyladenosine (m^6^A), 5-methylcytosine (m^5^C), and *N*^1^-methyladenosine (m^1^A) modifications in human, mouse, and rat demonstrates that HMB-Net consistently outperforms representative convolutional, recurrent, and Transformer-based baselines. Ablation experiments further confirm the benefit of the global-to-local encoding order over single-branch, reverse-ordered, and parallel dual-branch variants. Moreover, dimensionality reduction and integrated-gradient saliency analyses show that HMB-Net learns well-separated feature representations and autonomously focuses on conserved flanking consensus motifs (such as RRACH) without predefined biochemical rules, providing a reliable computational tool for transcriptome-wide modification mapping.

## Simple Summary

Inside living cells, ribonucleic acid molecules carry genetic instructions to produce proteins. Cells frequently attach small chemical groups, known as methyl groups, to specific positions on these molecules. These chemical modifications help control how genetic messages are processed, stabilized, and translated into proteins, and their abnormal regulation is closely associated with human diseases such as cancer and neurological disorders. Because experimental testing of every modified position across different tissues is costly and time-consuming, computational methods are needed to identify modified sites from sequence data. In this study, we developed a deep learning model that first captures broad sequence context and then refines local sequence patterns around each candidate site. We evaluated the model across twenty benchmark datasets covering three common types of ribonucleic acid methylation in human, mouse, and rat tissues. The model consistently achieved higher prediction accuracy than existing computational methods and transferred well across species. In addition, the model automatically identified known sequence patterns recognized by cellular modification enzymes. This work provides a practical and interpretable computational tool for studying gene regulation and disease mechanisms.

## 1. Introduction

RNA epitranscriptomics has reshaped our understanding of post-transcriptional gene regulation across eukaryotic systems [1,2]. To date, over 150 chemically diverse RNA modifications have been identified, which are dynamically deposited by “writers”, removed by “erasers”, and decoded by “readers”, thereby fine-tuning RNA splicing, translation efficiency, and transcript stability [3,4,5]. Beyond the widely studied *N*^6^-methyladenosine (m^6^A), accumulating evidence indicates that other modifications such as 5-methylcytosine (m^5^C) and *N*^1^-methyladenosine (m^1^A) also play important regulatory roles in cellular metabolism, host immunity, and tumor progression [6,7]. Experimentally, approaches such as antibody-based immunoprecipitation sequencing (e.g., MeRIP-seq and miCLIP) [8,9,10] and direct RNA nanopore sequencing [11] have advanced transcriptome-wide profiling of these marks. Nevertheless, wet-lab assays remain constrained by high experimental costs, antibody cross-reactivity, and stringent requirements on input RNA quantity.

To complement experimental profiling, computational prediction methods have evolved from traditional feature-engineered classifiers to end-to-end deep neural networks [12,13]. Early frameworks relied mainly on handcrafted sequence descriptors, such as pseudo-dinucleotide composition, paired with support vector machines (SVMs) or random forests [14,15,16], followed by gradient-boosted decision trees incorporating post-hoc feature attribution [17,18]. To reduce reliance on manual feature engineering, convolutional neural networks (CNNs) were introduced to detect localized motifs such as the canonical DRACH consensus [19,20], with residual architectures such as DeepPromise mitigating gradient vanishing in deeper networks [13]. Concurrently, recurrent neural networks (RNNs) and attention-augmented hybrids were developed to model sequential dependencies [21,22,23]. More recently, Transformer architectures and pretrained nucleotide language models have substantially advanced biological sequence modeling [24,25,26]. However, the quadratic complexity $O(L^{2})$ of standard self-attention incurs considerable computational overhead, whereas kernel-based linear attention approximations often lose resolution near motif boundaries [27]. Moreover, purely global sequence encoders can dilute the short, position-sensitive sequence signals characteristic of enzymatic modification sites. Conversely, while Selective State Space Models (SSMs) such as Mamba achieve linear-time O(L) scaling across long contexts [28,29], standalone state-space transitions may not fully resolve fine-grained local motif boundaries without dedicated local gating. In addition, existing hybrid architectures typically combine global and local extractors either in parallel or in a local-first cascade, where the local recurrent module processes raw nucleotide embeddings without prior global contextual filtering and can be misled by non-functional decoy motifs.

Another practical challenge in epitranscriptomic modeling lies in benchmark evaluation. Many existing predictors are evaluated on a single modification type or species, or on datasets without strict sequence identity filtering, which can inflate performance estimates due to homologous sequence leakage [30,31]. To address these architectural and evaluation challenges, we propose HMB-Net, an interpretable hybrid network that coordinates global contextual modeling with local motif refinement. Unlike parallel two-branch or local-first designs, HMB-Net employs a selective state space (Mamba) encoder to capture long-range sequence dependencies first, sequentially feeds these context-enriched representations into a multi-layer Bi-LSTM so that recurrent gates resolve local motif boundaries within a globally conditioned latent space, and integrates both representations via bidirectional cross-attention. We systematically evaluated HMB-Net across 20 non-redundant mammalian benchmark datasets covering m^6^A, m^5^C, and m^1^A modifications compiled from tissue-specific profiles, RMBase, and m6A-Atlas [31,32,33], including comparisons against single-branch, reverse-ordered, and parallel dual-branch variants as well as cross-species transfer tests. Furthermore, representation visualization and integrated-gradient attribution analyses [34] demonstrate that HMB-Net captures biologically meaningful consensus motifs directly from primary RNA sequences.

## 2. Materials and Methods

### 2.1. Benchmark Datasets

To evaluate the generalization performance of HMB-Net, we compiled a benchmark suite comprising 20 datasets from three established resources: Dao et al. [31], RMBase v2.0 [32], and m6A-Atlas [33]. This collection spans three mammalian species (*Homo sapiens*, *Mus musculus*, and *Rattus norvegicus*) and three representative RNA methylation types (m^6^A, m^5^C, and m^1^A). The benchmark is organized into two evaluation tracks. The first track examines tissue-specific m^6^A prediction using 11 high-resolution datasets from Dao et al. [31], covering brain, liver, kidney, heart, and testis across human, mouse, and rat. The second track evaluates cross-modification and cross-platform generalization using 9 datasets from RMBase and m6A-Atlas spanning m^6^A, m^5^C, and m^1^A modifications.

To prevent data leakage from sequence homology, sequence redundancy was removed globally using CD-HIT [30] with an identity cutoff of 80% prior to splitting the training and independent testing sets, ensuring that no two sequences across the training and test subsets share more than 80% sequence similarity. Positive and negative samples were maintained at a balanced 1:1 ratio, and the non-redundant sequences were partitioned into disjoint training and independent testing sets as summarized in Table 1. Within each dataset, models were optimized using stratified 5-fold cross-validation on the training set and evaluated on the isolated independent testing set without cross-dataset pooling, thereby avoiding sequence overlap between training and testing sets across related datasets.

### 2.2. Overview of the HMB-Net Framework

**Figure 1:**
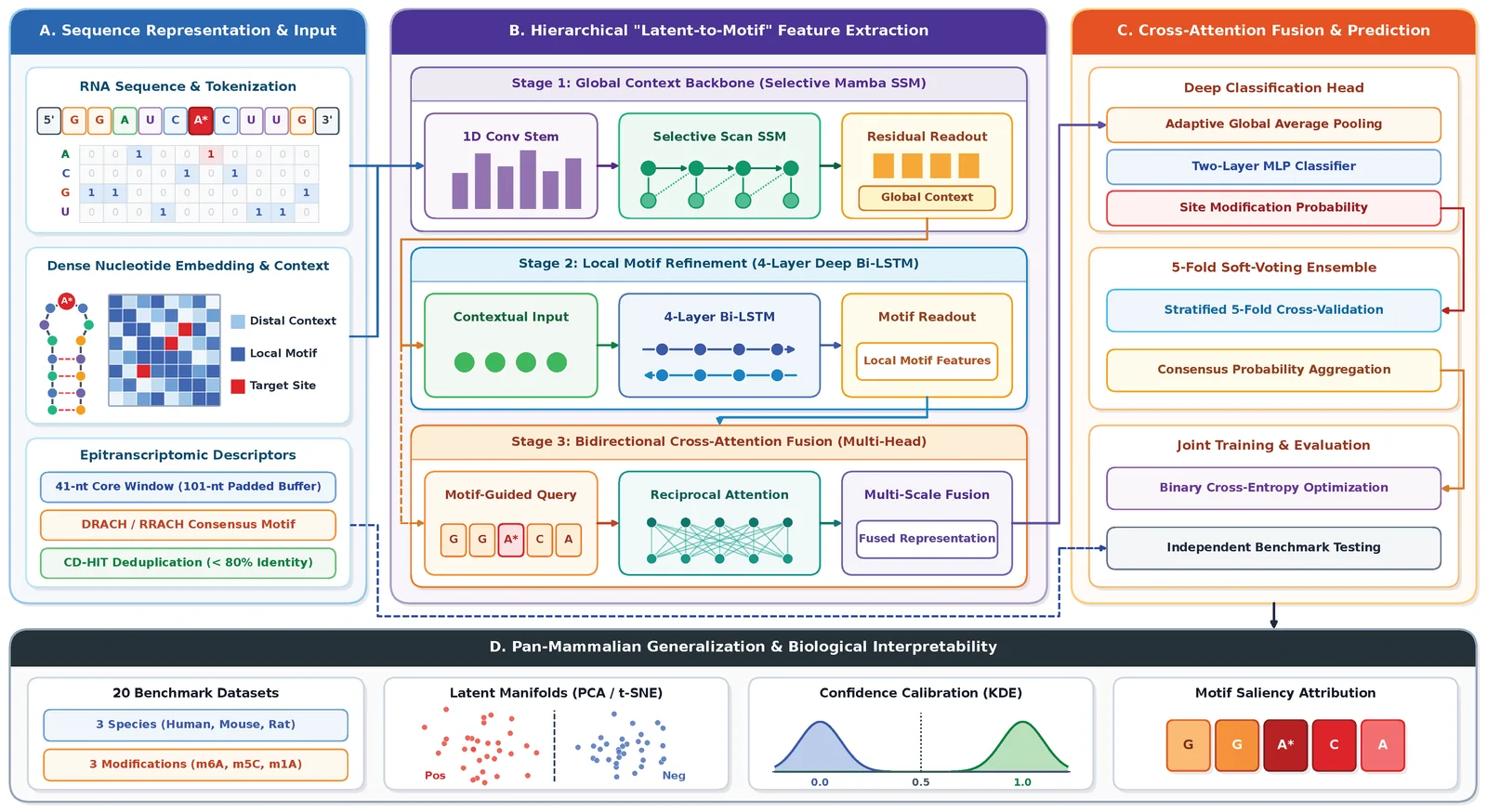
Schematic overview of the HMB-Net framework. (**A**) Sequence Representation and Input: RNA sequence windows (41-nt core sequence window within a 101-nt padded input buffer) centered on the candidate site are tokenized and projected into dense nucleotide embeddings without external secondary-structure descriptors. (**B**) Hierarchical “Latent-to-Motif” Feature Extraction: Stage 1 models long-range sequence context via a Selective Mamba SSM backbone, Stage 2 refines local motif features via a four-layer deep Bi-LSTM, and Stage 3 integrates global and local representations via multi-head bidirectional cross-attention. (**C**) Cross-Attention Fusion and Prediction: adaptive global average pooling, two-layer MLP classification, and 5-fold soft-voting ensemble inference. (**D**) Pan-Mammalian Generalization and Biological Interpretability across 20 benchmark datasets spanning three mammalian species and three modification types. Arrows indicate the direction of forward information flow, the asterisk (A*) marks the central candidate modification site, and distinct colors denote different functional modules.

To capture both distal sequence context and local motif patterns for RNA methylation site identification, we developed the Hierarchical Mamba-BiLSTM Network (HMB-Net). As illustrated in Figure 1, the overall workflow of HMB-Net is organized into four panels: (A) Sequence Representation and Input, which encodes centered RNA sequence windows from non-redundant datasets into discrete tokens and dense nucleotide embedding representations; (B) Hierarchical “Latent-to-Motif” Feature Extraction, where Stage 1 employs a Selective State Space Model (Mamba) backbone to capture global sequence context, Stage 2 sequentially feeds the context-aware representations into a four-layer Bi-LSTM to refine local motif boundaries, and Stage 3 applies multi-head bidirectional cross-attention to fuse both scales; (C) Cross-Attention Fusion and Prediction, which aggregates the fused features via adaptive global average pooling, estimates site-level modification probabilities through a two-layer MLP classification head, and integrates stratified 5-fold models via soft-voting consensus; and (D) Pan-Mammalian Generalization and Biological Interpretability across 20 benchmark datasets, encompassing latent manifold projection (PCA/t-SNE), confidence calibration (KDE), and base-resolution motif saliency attribution.

### 2.3. Input Representation

Formally, we define an input RNA sequence segment as $S=(n_{1},n_{2},\ldots ,n_{L})$, where L=101 denotes the fixed input buffer length accommodating the 41-nt core sequence window (±20 nt centered on the candidate modification site at position 21, with trailing positions 42–101 zero-padded), and each nucleotide $n_{i}$ belongs to the vocabulary V={N/PAD,A,U/T,G,C} indexed from 0 to 4. The objective is to learn a mapping function $f:S\to \hat{y}\in \left[0,1\right]$ representing the probability that the central nucleotide is methylated. Each discrete token is projected into a continuous *d*-dimensional latent space (d=128) via a learnable embedding matrix $W_{e}\in R^{|V|\times d}$ (with padding index 0 mapped to a zero vector), yielding the embedded sequence representation $X=\mathrm{Embedding}\left(S\right)\in R^{L\times d}$. The architecture uses these primary nucleotide embeddings alone and does not incorporate additional secondary-structure descriptors.

### 2.4. Global Context Modeling via Selective State Space Model

To model long-range sequence dependencies with O(L) linear complexity, we employ a Selective State Space Model (Mamba) block [28]. After Zero-Order Hold (ZOH) discretization with an input-dependent step size Δ, the continuous state transition matrix $A\in R^{d_{\mathrm{state}}\times d_{\mathrm{state}}}$ ($d_{\mathrm{state}}=16$) and input projection $B\in R^{d_{\mathrm{state}}\times 1}$ are transformed into discrete parameters $\bar{A}=\exp\left(\Delta A\right)$ and $\bar{B}={\left(\Delta A\right)}^{-1}\left(\exp\left(\Delta A\right)-I\right)\Delta B$. At sequence position *t*, the selective state space recurrence is computed as:

(1) $$\begin{array}{cc} h_{t} & =\bar{A}h_{t-1}+\bar{B}x_{t},\\ y_{t} & =Ch_{t}, \end{array}$$

where $C\in R^{1\times d_{\mathrm{state}}}$ is the output projection. By parameterizing Δ, B, and C as functions of the input sequence X (with local convolution width $d_{\mathrm{conv}}=4$ and expansion factor 2), the Mamba block selectively propagates informative distal signals while filtering background sequence noise. The global contextual representation $H_{M}\in R^{L\times d}$ is then obtained through a residual connection followed by layer normalization (LN):

(2) $$H_{M}=\mathrm{LN}\left(\mathrm{SSM}\left(X\right)+X\right).$$

### 2.5. Local Motif Refinement via Deep Bi-LSTM

While the Mamba encoder captures broad contextual dependencies across the 101-nt window, recurrent gating mechanisms are effective at resolving sharp local motif boundaries around the methylation site. In our cascaded design, $H_{M}$ is fed directly into a four-layer Bidirectional LSTM (Bi-LSTM) network with hidden dimension $d_{h}=64$ per direction and inter-layer dropout of 0.3. At sequence position *t*, the forward and backward hidden states of the top Bi-LSTM layer are updated as:

(3) $$\begin{array}{cc} {\vec{h}}_{t} & ={\mathrm{LSTM}}_{\mathrm{fwd}}\left({\vec{h}}_{t-1},H_{M}\left[t,:\right]\right),\\ {\overset{\leftarrow }{h}}_{t} & ={\mathrm{LSTM}}_{\mathrm{bwd}}\left({\overset{\leftarrow }{h}}_{t+1},H_{M}\left[t,:\right]\right). \end{array}$$

Concatenating the bidirectional hidden states $h_{t}=\left[{\vec{h}}_{t}\;;\;{\overset{\leftarrow }{h}}_{t}\right]\in R^{d}$ ($2d_{h}=d=128$) across all positions and applying layer normalization yields the locally refined representation $H_{L}\in R^{L\times d}$:

(4) $$H_{L}=\mathrm{LN}\left(\mathrm{Bi-LSTM}\left(H_{M}\right)\right)=\mathrm{LN}\left({\left[h_{1},h_{2},\ldots ,h_{L}\right]}^{⊤}\right).$$

Through this sequential cascade, $H_{L}$ preserves the long-range contextual prior encoded in $H_{M}$ while sharpening local sequence representations around candidate motifs.

### 2.6. Bidirectional Cross-Attention Fusion

To integrate the global context representation ($H_{M}$) and the locally refined representation ($H_{L}$), we employ a Bidirectional Cross-Attention module with h=4 attention heads (per-head dimension $d_{k}=d/h=32$). Using scaled dot-product attention $\mathrm{Attn}\left(Q,K,V\right)=\mathrm{softmax}\left(QK^{⊤}/\sqrt{d_{k}}\right)V$, we construct two reciprocal cross-attention pathways:

(5) $$\begin{array}{cc} Z_{1} & =\mathrm{Attn}(H_{L}W_{Q}^{\left(1\right)},H_{M}W_{K}^{\left(1\right)},H_{M}W_{V}^{\left(1\right)}), \end{array}$$

(6) $$\begin{array}{cc} Z_{2} & =\mathrm{Attn}(H_{M}W_{Q}^{\left(2\right)},H_{L}W_{K}^{\left(2\right)},H_{L}W_{V}^{\left(2\right)}), \end{array}$$

where $W_{Q}^{\left(i\right)},W_{K}^{\left(i\right)},W_{V}^{\left(i\right)}\in R^{d\times d}$ (i∈{1,2}) are learnable projection matrices. The fused representation $H_{F}\in R^{L\times d}$ is then formed via multi-scale residual addition and layer normalization:

(7) $$H_{F}=\mathrm{LN}\left(Z_{1}+Z_{2}+H_{L}+H_{M}\right).$$

### 2.7. Sequence-Level Prediction Layer

To aggregate position-wise features into a sequence-level descriptor, $H_{F}$ is summarized along the sequence dimension via adaptive global average pooling, $v=\frac{1}{L}\sum_{t=1}^{L}H_{F}\left[t,:\right]\in R^{d}$. The pooled vector v is then passed through a two-layer MLP classification head (128→64→1) with ReLU activation and dropout (p=0.3) to estimate the site modification probability:

(8) $$\hat{y}=\sigma \left(W_{2}\;\mathrm{Dropout}\left(\mathrm{ReLU}\left(W_{1}v+b_{1}\right)\right)+b_{2}\right),$$

where $W_{1}\in R^{64\times 128}$, $b_{1}\in R^{64}$, $W_{2}\in R^{1\times 64}$, $b_{2}\in R$, and σ(·) denotes the logistic sigmoid function. The network is trained end-to-end by minimizing the binary cross-entropy loss using the Adam optimizer with an initial learning rate of $1.0\times 10^{-3}$ and a weight decay of $1.0\times 10^{-4}$ for up to 50 epochs.

### 2.8. Model Ensemble and Independent Validation

To reduce variance from individual data splits and improve prediction stability on unseen sequences, we adopt a soft voting ensemble strategy during independent testing. Specifically, on each training dataset, we perform stratified *K*-fold cross-validation (K=5), yielding *K* trained model instances $M=\{M_{1},M_{2},\ldots ,M_{K}\}$ with corresponding parameter sets $\left\{\theta_{1},\theta_{2},\ldots ,\theta_{K}\right\}$. When evaluating an independent test sample $S_{i}$, the final predicted probability ${\hat{y}}_{i}$ is computed as the arithmetic mean of the probability estimates produced by all *K* models:

(9) $${\hat{y}}_{i}=\frac{1}{K}\sum_{k=1}^{K}P\left(y_{i}=1|S_{i};\theta_{k}\right),$$

where $P(y_{i}=1|S_{i};\theta_{k})$ denotes the sigmoid output of the *k*-th fold model $M_{k}$. Averaging the predicted probability distributions across folds mitigates fold-specific overfitting and yields more reliable decision boundaries on the independent test sets.

### 2.9. Performance Evaluation Metrics

We evaluated model performance using six threshold-dependent metrics derived from the confusion matrix: Accuracy (ACC), Sensitivity (Sn), Specificity (Sp), Precision (Pre), F1-score (F1), and Matthews Correlation Coefficient (MCC). Let TP, TN, FP, and FN denote the numbers of true positives, true negatives, false positives, and false negatives, respectively. These metrics are defined as follows:

(10) $$\begin{array}{c} \mathrm{Accuracy}\left(\mathrm{ACC}\right)=\frac{\mathrm{TP}+\mathrm{TN}}{\mathrm{TP}+\mathrm{TN}+\mathrm{FP}+\mathrm{FN}},\\ \mathrm{Sensitivity}\left(\mathrm{Sn}\right)=\frac{\mathrm{TP}}{\mathrm{TP}+\mathrm{FN}},\\ \mathrm{Specificity}\left(\mathrm{Sp}\right)=\frac{\mathrm{TN}}{\mathrm{TN}+\mathrm{FP}},\\ \mathrm{Precision}\left(\mathrm{Pre}\right)=\frac{\mathrm{TP}}{\mathrm{TP}+\mathrm{FP}},\\ F\mathrm{1-score}=\frac{2\times \mathrm{Pre}\times \mathrm{Sn}}{\mathrm{Pre}+\mathrm{Sn}},\\ \mathrm{MCC}=\frac{\mathrm{TP}\times \mathrm{TN}-\mathrm{FP}\times \mathrm{FN}}{\sqrt{\left(\mathrm{TP}+\mathrm{FP})(\mathrm{TP}+\mathrm{FN})(\mathrm{TN}+\mathrm{FP})(\mathrm{TN}+\mathrm{FN}\right)}}. \end{array}$$

In addition to fixed-threshold metrics (evaluated at a classification threshold of 0.5), we computed the Area Under the Receiver Operating Characteristic Curve (ROC-AUC) and the Area Under the Precision-Recall Curve (PR-AUC) to assess threshold-independent discrimination across operating points. To assess prediction stability and statistical significance, we also report the mean and standard deviation (Mean ± SD) across the five cross-validation folds and perform two-sided paired Wilcoxon signed-rank tests and paired Student’s *t*-tests across the 20 benchmark datasets.

## 3. Results

### 3.1. Baseline Models

To evaluate HMB-Net across different architectural paradigms, we compared it against three representative deep learning predictors spanning convolutional, hybrid recurrent, and Transformer-based designs. First, DeepPromise [13] serves as a CNN-based baseline; originally developed for m^1^A and m^6^A prediction, it employs deep residual convolutional blocks with skip connections to extract multi-scale local sequence features. Second, DeepMRMP [23] represents a hybrid recurrent architecture that combines 1D convolutional layers with a Bidirectional LSTM (Bi-LSTM) to capture local patterns alongside bidirectional sequence dependencies. Third, M6A-BERT [26] represents a Transformer-based architecture utilizing multi-head self-attention to model global nucleotide interactions across the input window. For a fair comparison, all baseline architectures and HMB-Net were configured with comparable model capacities (∼0.42M–0.66M trainable parameters) and trained and evaluated on the exact same 5-fold cross-validation splits using identical optimization settings (Adam optimizer, learning rate $1.0\times 10^{-3}$, weight decay $1.0\times 10^{-4}$, batch size 128, up to 50 epochs with early stopping) and the same 5-fold soft-voting ensemble strategy in Equation (9). In addition, single-fold HMB-Net models evaluated on the independent test sets exhibit low fold-to-fold variance (${\mathrm{SD}}_{\mathrm{ACC}}\in \left[0.0011,0.0150\right]$; e.g., ACC=0.7307±0.0046 on h_b, 0.7995±0.0082 on h_l, and 0.8282±0.0022 on r_k) and already match or outperform the 5-fold ensembled baselines, confirming that the performance gains are not merely attributable to ensembling or differences in model capacity.

### 3.2. Comparison with Existing Methods on Tissue-Specific Datasets

We first benchmarked HMB-Net against DeepMRMP, DeepPromise, and M6A-BERT across the 11 tissue-specific m^6^A datasets from Dao et al. [31], covering human, mouse, and rat tissues. As shown in Table 2, HMB-Net consistently achieves the highest performance across all 11 tissue benchmarks in terms of ACC, Sn, Sp, Pre, F1-score, MCC, and ROC-AUC. For example, on the human kidney (h_k) independent test set, HMB-Net attains an ACC of 0.7897, an MCC of 0.5808, and an ROC-AUC of 0.8807, outperforming M6A-BERT (ACC: 0.7760, MCC: 0.5529, ROC-AUC: 0.8630) and DeepMRMP (ACC: 0.7644, MCC: 0.5289, ROC-AUC: 0.8370). Moreover, while baselines often exhibit a trade-off between sensitivity and specificity, HMB-Net improves both metrics simultaneously on h_k (Sn: 0.7538, Sp: 0.8255). Similar improvements are observed on the rodent benchmarks. On the rat kidney (r_k) dataset, HMB-Net reaches an F1-score of 0.8413 and an MCC of 0.6748, surpassing DeepMRMP (F1: 0.8267, MCC: 0.6481) and DeepPromise (F1: 0.8071, MCC: 0.6140). Even on more challenging datasets such as mouse liver (m_l), HMB-Net improves MCC from 0.4015 (DeepMRMP) to 0.4649 and ROC-AUC from 0.7624 to 0.8100, indicating robust discrimination of tissue-specific methylation signals from background sequences. Across all 20 benchmark datasets, paired Wilcoxon signed-rank tests confirm that the improvements of HMB-Net over DeepMRMP ($p=1.91\times 10^{-6}$), DeepPromise ($p=1.91\times 10^{-6}$), and M6A-BERT ($p=8.84\times 10^{-5}$ for ACC and $p=1.91\times 10^{-6}$ for MCC and ROC-AUC) are statistically significant.

### 3.3. Evaluation of Cross-Modification and Cross-Platform Generalization

To evaluate whether HMB-Net generalizes beyond tissue-specific m^6^A profiles, we tested it on 9 cross-modification and cross-platform datasets derived from RMBase v2.0 and m6A-Atlas, covering m^6^A, m^5^C, and m^1^A across human, mouse, and rat.

**Figure 2:**
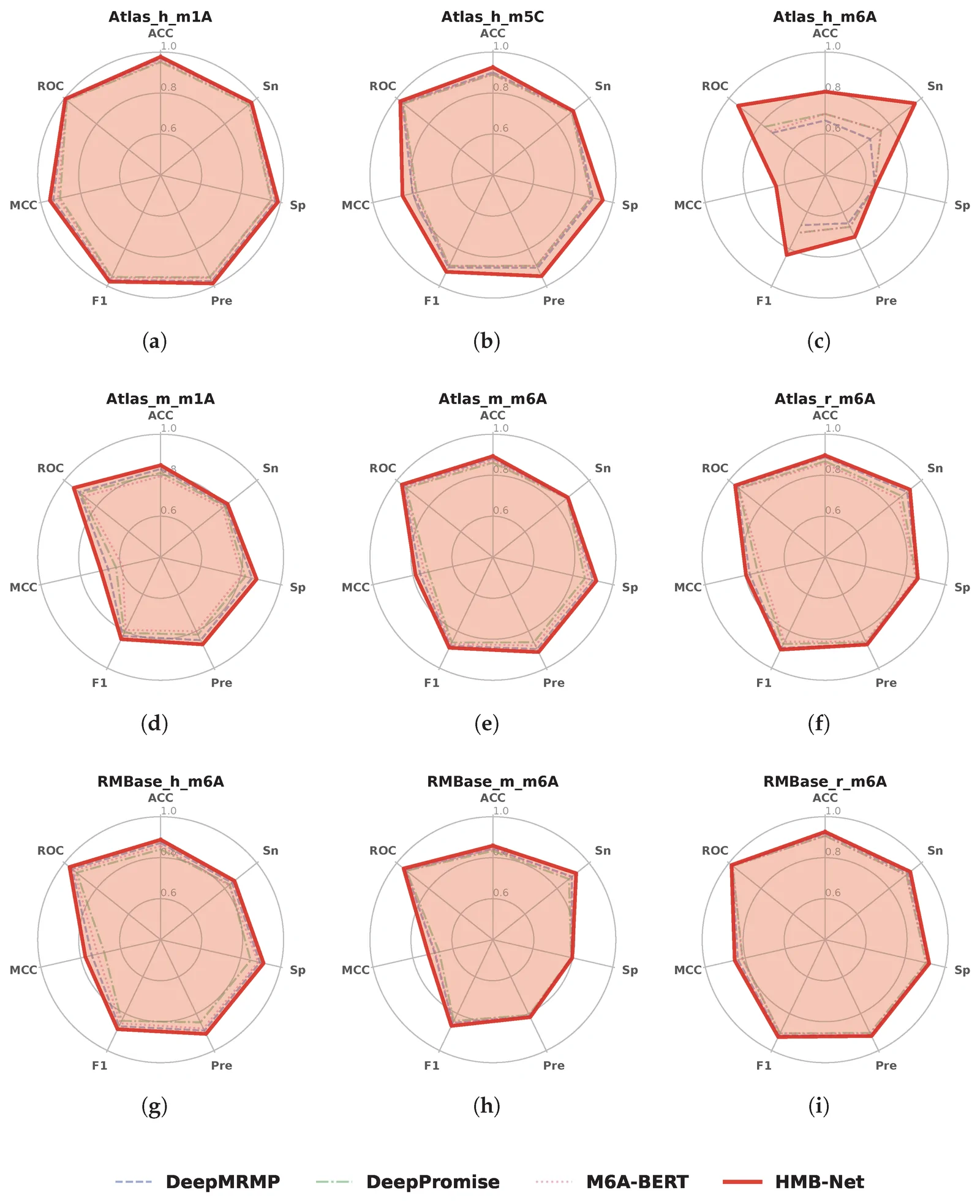
Radar-chart comparison of HMB-Net and baseline methods across 9 datasets from m6A-Atlas and RMBase v2.0 spanning human, mouse, and rat (m^1^A, m^5^C, and m^6^A). Panels (**a**–**i**) correspond to each benchmark dataset. HMB-Net (solid red line) achieves well-balanced performance across evaluation metrics relative to the baseline models (dashed lines).

**Figure 3:**
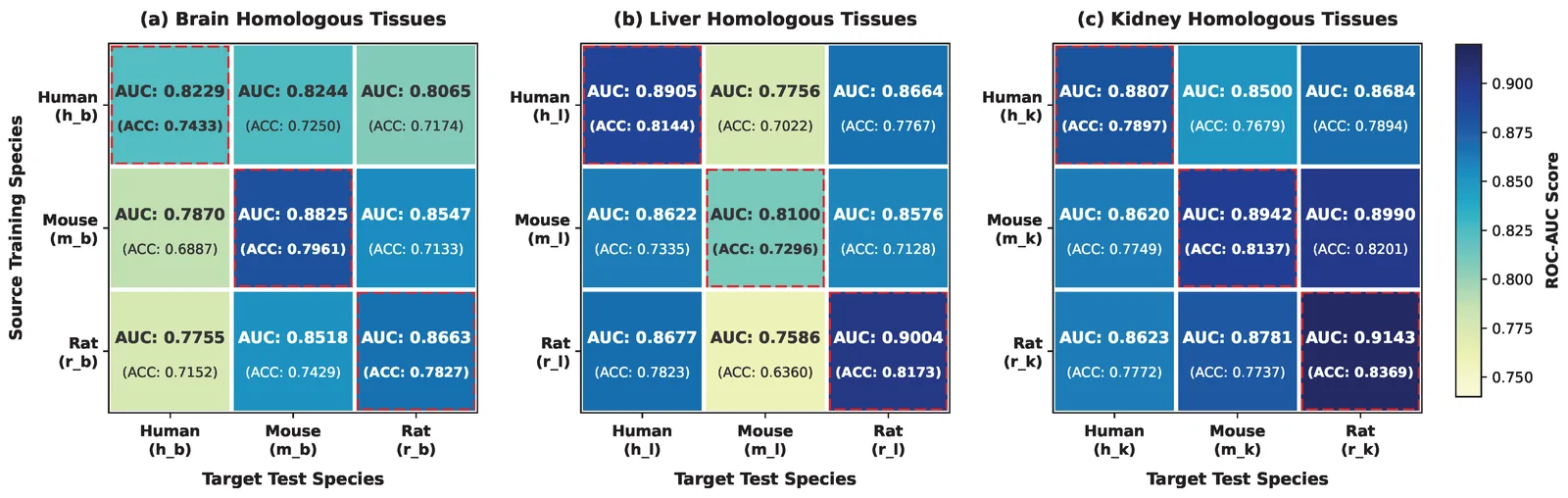
Cross-species zero-shot transfer heatmaps of HMB-Net across human, mouse, and rat in three homologous tissues: (**a**) brain (h_b, m_b, r_b), (**b**) liver (h_l, m_l, r_l), and (**c**) kidney (h_k, m_k, r_k). Diagonal cells (red dashed boxes) denote within-species performance, while off-diagonal cells denote zero-shot cross-species transfer ROC-AUC and accuracy (ACC) without retraining.

As shown in the radar charts of Figure 2 (Panels a to i), HMB-Net (solid red line) consistently encloses the baseline profiles across the evaluated metrics. For example, on the human m^1^A (Atlas_h_m1A, Figure 2a) and human m^5^C (Atlas_h_m5C, Figure 2b) benchmarks, HMB-Net achieves the highest accuracy (0.9773 and 0.9259), F1-score (0.9770 and 0.9240), specificity (0.9873 and 0.9503), and ROC-AUC (0.9954 and 0.9779). Similarly, on the cross-platform m^6^A datasets from m6A-Atlas and RMBase (Figure 2c,e–i), HMB-Net maintains balanced sensitivity and specificity without the metric skew observed in individual baselines. The complete numerical results corresponding to Figure 2 are provided in Table S1. In addition, to examine cross-species transferability without retraining, we evaluated the pairwise zero-shot transfer performance of HMB-Net across human, mouse, and rat in three homologous tissues: brain (h_b, m_b, r_b), liver (h_l, m_l, r_l), and kidney (h_k, m_k, r_k). As shown in the cross-species transfer heatmaps of Figure 3, models trained on one mammalian species generalize well to unseen test sets of the other two species across all three homologous tissues (for example, human-trained models achieve zero-shot ROC-AUCs of 0.8244 on h_b → m_b, 0.8065 on h_b → r_b, 0.7756 on h_l → m_l, 0.8664 on h_l → r_l, 0.8500 on h_k → m_k, and 0.8684 on h_k → r_k, while mouse- and rat-trained models achieve up to 0.8990 on m_k → r_k and 0.8781 on r_k → m_k).

### 3.4. Ablation Study and Architectural Analysis

To examine the contribution of each architectural module and test the rationale behind our “Latent-to-Motif” hierarchy, we conducted an ablation study across all 20 benchmark datasets. We compared the complete HMB-Net against four ablated variants: (1) BiLSTMOnly, which removes the Mamba block and relies solely on the four-layer Bi-LSTM to evaluate performance without global state-space context modeling; (2) MambaOnly, which removes the Bi-LSTM branch and relies solely on the Mamba encoder to evaluate whether global state-space transitions alone suffice for site-level prediction; (3) R-HMB-Net (Reverse Order), which reverses the sequential encoding order (Input→Bi-LSTM→Mamba) to test whether extracting global context prior to local motif refinement is advantageous; and (4) P-HMB-Net (Parallel Order), which processes the nucleotide embeddings through the Mamba and four-layer Bi-LSTM branches in parallel before bidirectional cross-attention fusion.

#### 3.4.1. Impact of Individual Encoding Components

**Figure 4:**
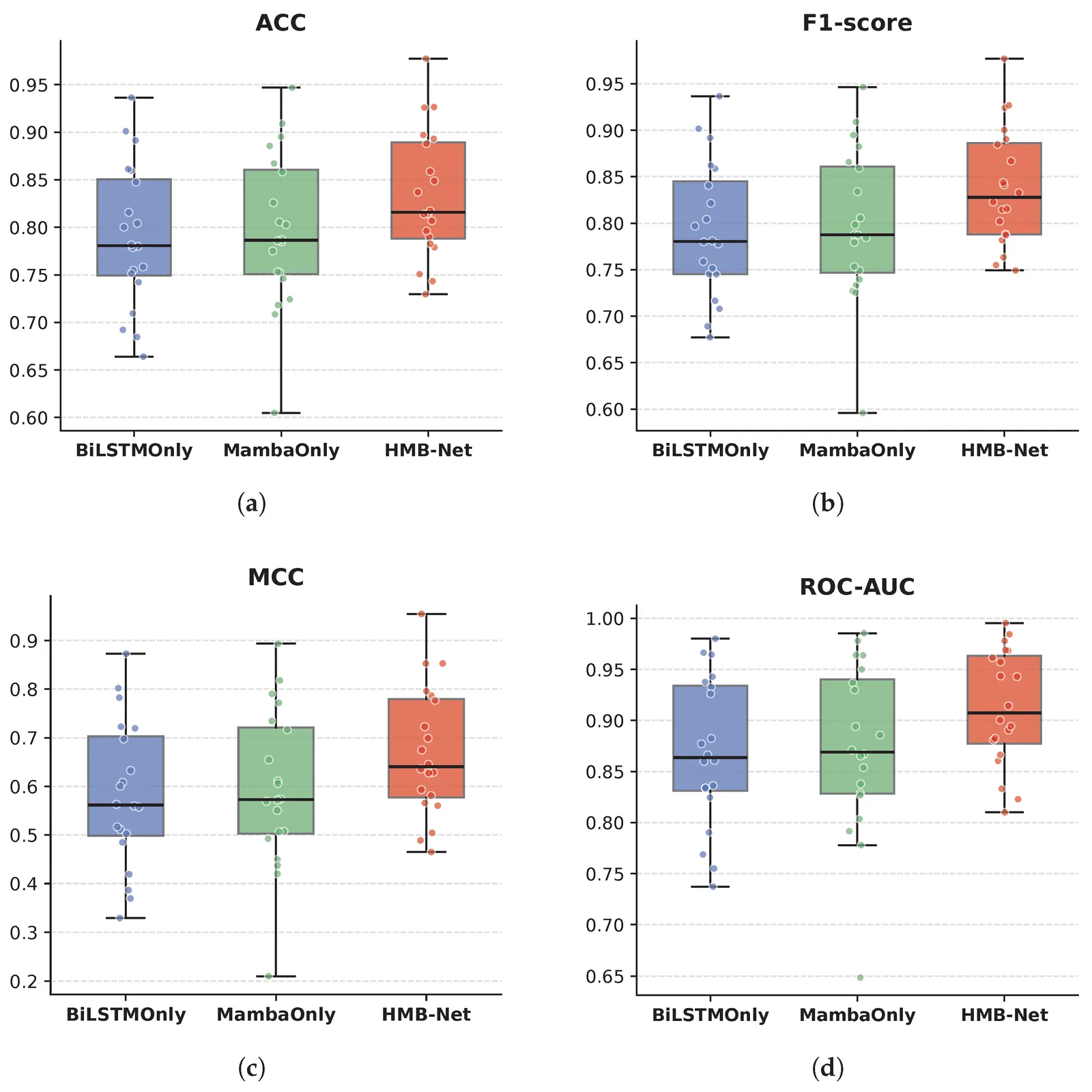
Boxplot comparison of HMB-Net against single-branch ablation variants (BiLSTMOnly and MambaOnly) across 20 benchmark datasets on (**a**) ACC, (**b**) F1-score, (**c**) MCC, and (**d**) ROC-AUC.

Figure 4 compares the metric distributions of HMB-Net against the single-branch BiLSTMOnly and MambaOnly variants across all 20 datasets. The boxplots show that HMB-Net achieves higher median values and tighter interquartile ranges across ACC, F1-score, MCC, and ROC-AUC ($p=1.91\times 10^{-6}$, paired Wilcoxon signed-rank test). In particular, the MambaOnly variant exhibits larger performance variance on noisier tissue datasets, indicating that global state-space representations benefit from explicit local gating to resolve motif boundaries. Conversely, BiLSTMOnly shows lower overall accuracy and MCC, reflecting the difficulty of capturing long-range flanking context with recurrent gating alone. Combining both modules in HMB-Net reduces false positives while preserving high sensitivity across diverse sequence contexts.

#### 3.4.2. Analysis of Sequential Ordering: Latent-to-Motif vs. Motif-to-Latent

**Figure 5:**
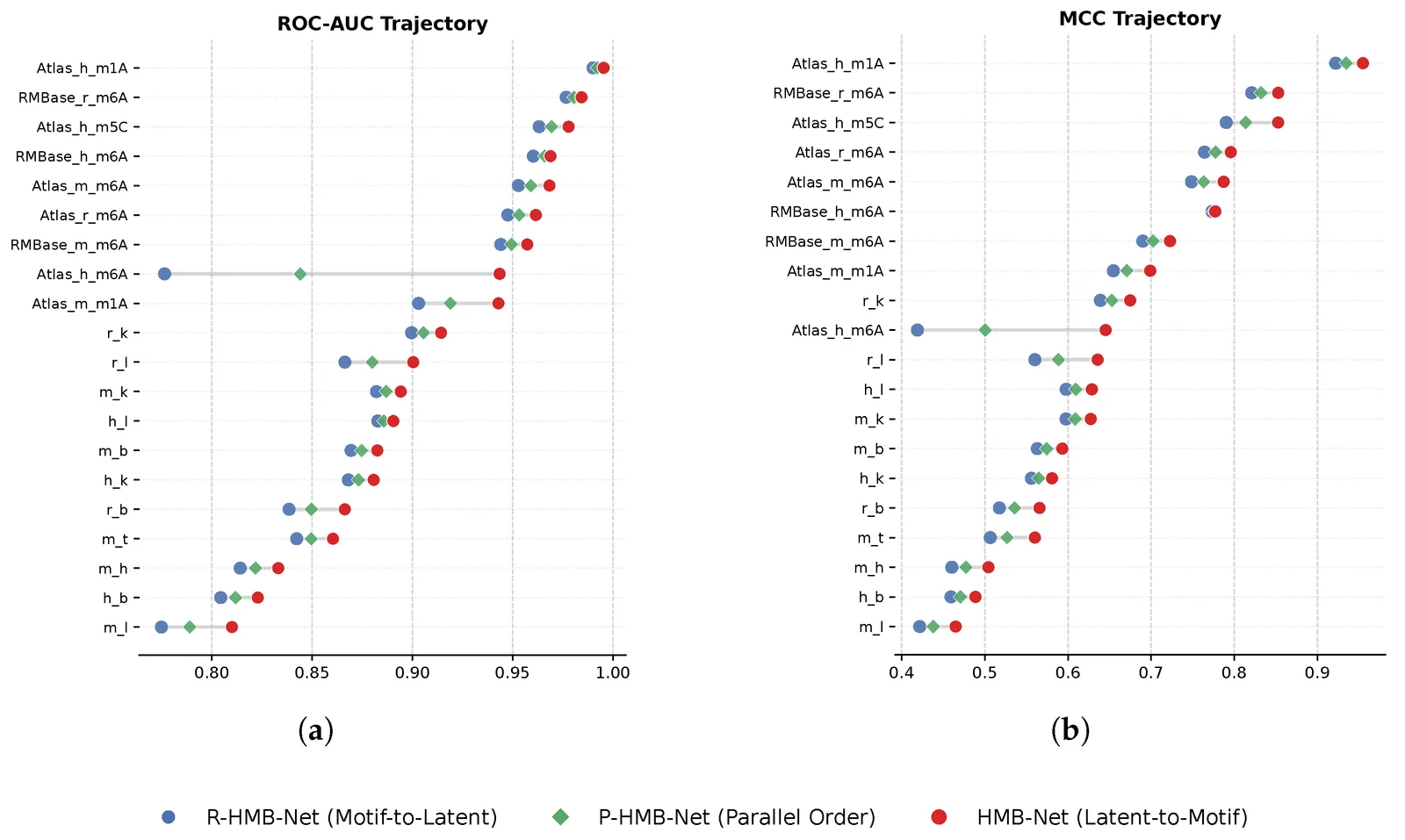
Effect of module ordering and coupling topology across 20 benchmark datasets. The dumbbell plots compare the reverse Motif-to-Latent variant (R-HMB-Net, blue dots), the parallel dual-branch variant (P-HMB-Net, green diamonds), and the proposed Latent-to-Motif architecture (HMB-Net, red dots) in terms of (**a**) ROC-AUC and (**b**) MCC.

In a hybrid network, the coupling topology of functional modules determines how sequence information is progressively refined. Figure 5 uses a dumbbell plot to compare the reverse “Motif-to-Latent” configuration (R-HMB-Net, blue dots), the parallel dual-branch configuration (P-HMB-Net, green diamonds), and our sequential “Latent-to-Motif” configuration (HMB-Net, red dots) across all 20 datasets. HMB-Net consistently achieves higher ROC-AUC and MCC than both R-HMB-Net and P-HMB-Net across all 20 datasets ($p\leq 8.84\times 10^{-5}$, Wilcoxon signed-rank test). On challenging tissue benchmarks (such as m_l and r_l), applying the Bi-LSTM directly to raw token embeddings in R-HMB-Net makes the model more susceptible to local sequence fluctuations, which then propagate into the subsequent Mamba block. While P-HMB-Net improves over R-HMB-Net by extracting Mamba and Bi-LSTM representations in parallel, its Bi-LSTM branch still processes uncontextualized nucleotide embeddings without prior global filtering. By contrast, HMB-Net uses the Mamba block first to establish a smoothed global context representation before the Bi-LSTM refines local motif features. Full numerical results for all five ablation variants across the 20 datasets are reported in Table S2.

### 3.5. Model Interpretability and Visual Analysis

To understand the internal representations and decision criteria learned by HMB-Net, we performed dimensionality reduction, prediction confidence analysis, and gradient-based sequence attribution across multiple benchmark datasets.

#### 3.5.1. Feature Representation Analysis

**Figure 6:**
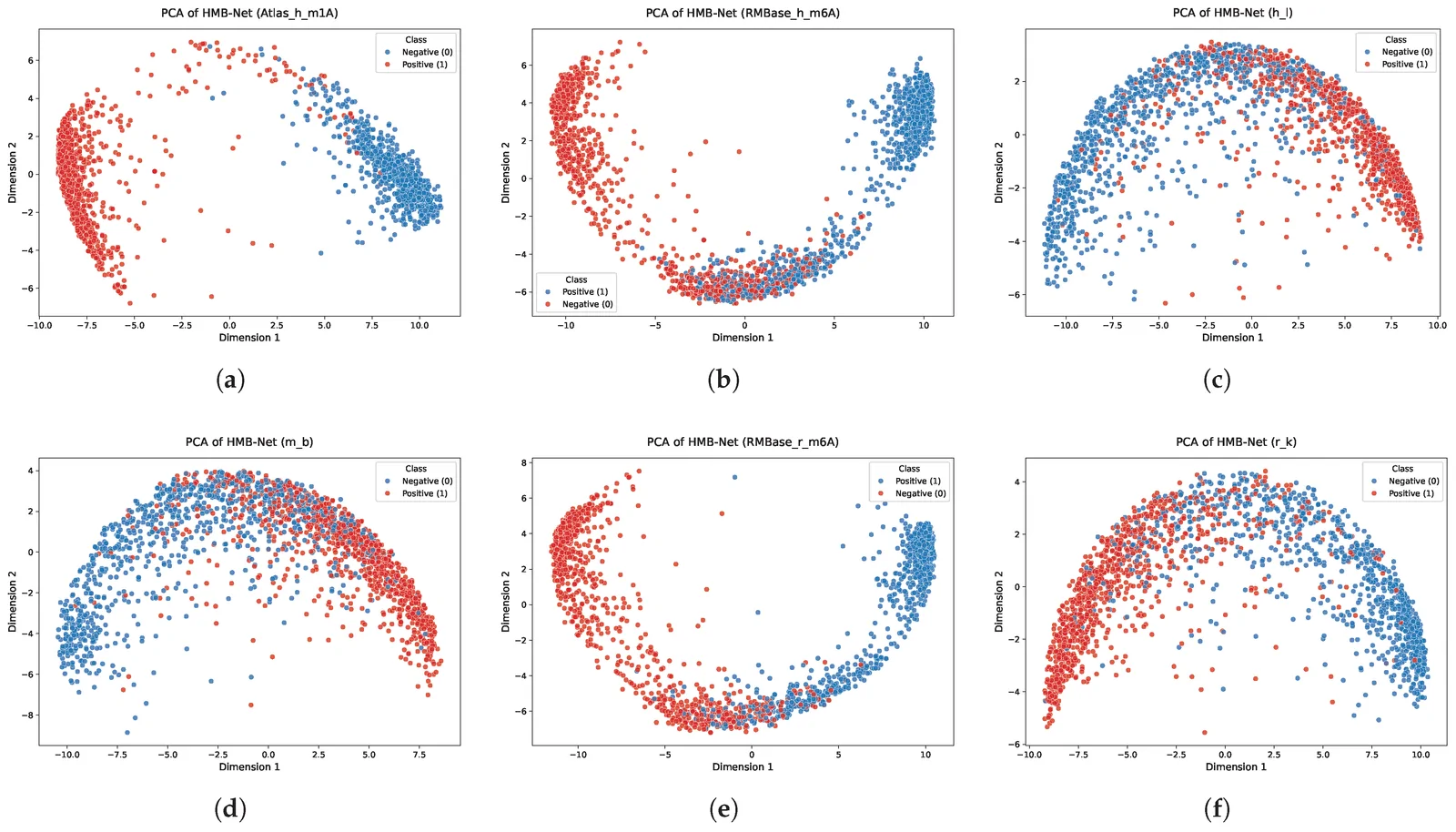
Principal Component Analysis (PCA) projections of learned feature representations across six representative datasets: (**a**) Atlas_h_m1A, (**b**) RMBase_h_m6A, (**c**) h_l, (**d**) m_b, (**e**) RMBase_r_m6A, and (**f**) r_k.

**Figure 7:**
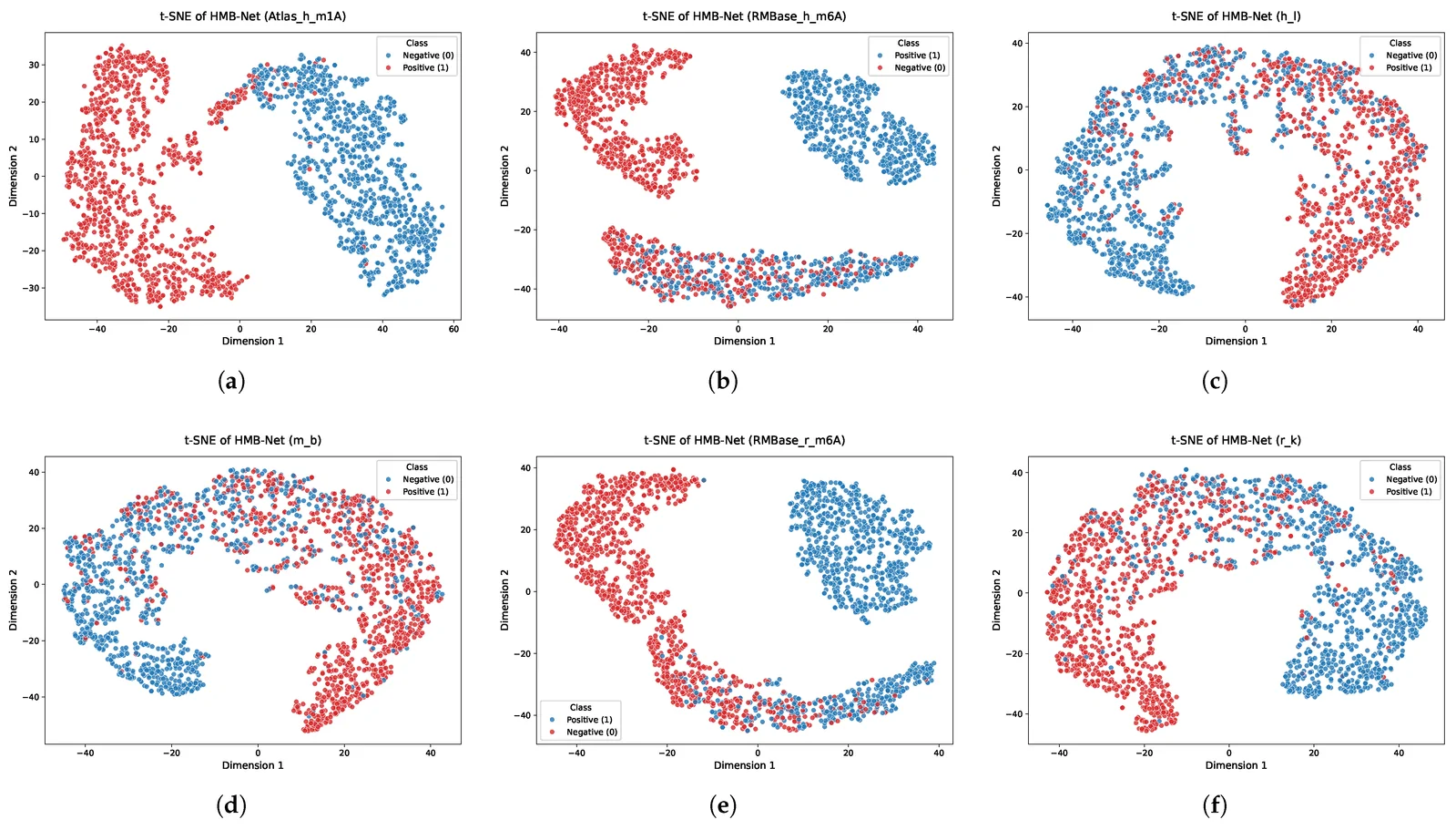
t-SNE projections of learned sequence representations on the same six representative datasets: (**a**) Atlas_h_m1A, (**b**) RMBase_h_m6A, (**c**) h_l, (**d**) m_b, (**e**) RMBase_r_m6A, and (**f**) r_k.

To examine how HMB-Net separates methylated from non-methylated sequences in its latent space, we projected the learned feature vectors prior to the final classification layer into two dimensions. Figure 6 shows the Principal Component Analysis (PCA) projections across six representative datasets (Atlas_h_m1A, RMBase_h_m6A, h_l, m_b, RMBase_r_m6A, and r_k), revealing clear separation between positive and negative samples along the primary variance axes. To further inspect nonlinear structure, Figure 7 presents the corresponding t-Distributed Stochastic Neighbor Embedding (t-SNE) projections on the same six datasets. The embeddings form two well-defined clusters corresponding to methylated and non-methylated sites with limited overlap across human, mouse, and rat benchmarks.

#### 3.5.2. Prediction Confidence Density

**Figure 8:**
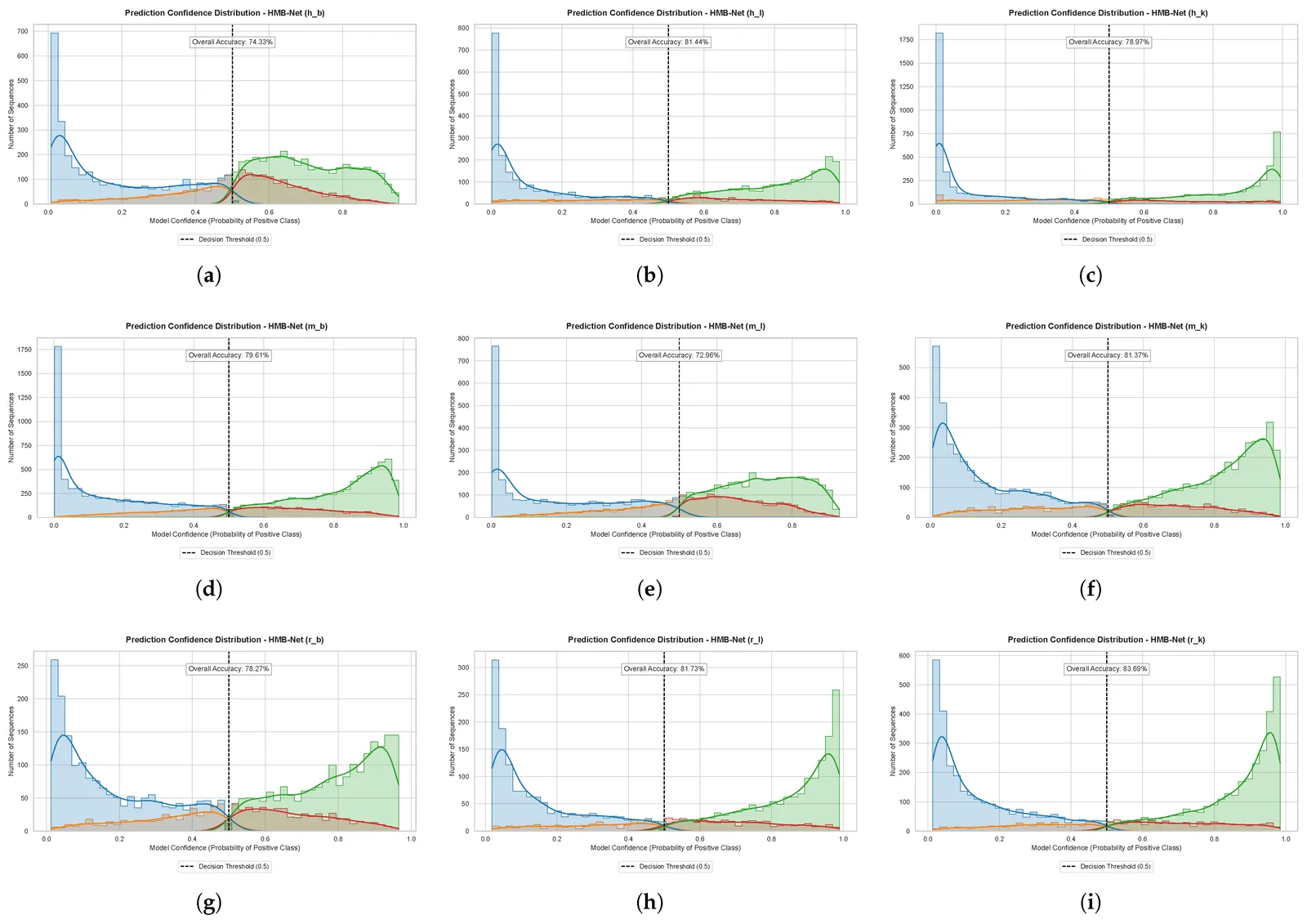
Kernel density distributions of predicted probabilities across nine tissue datasets (**a**–**i**) (brain, liver, and kidney in human, mouse, and rat). The bimodal distributions show that predictions concentrate near 0 and 1 with low uncertainty around the 0.5 threshold. Different colors represent the nine individual tissue benchmark datasets, and the vertical dashed line indicates the 0.5 classification threshold.

In addition to thresholded classification accuracy, the distribution of predicted probabilities reflects model calibration and decision certainty. Figure 8 displays the Kernel Density Estimation (KDE) profiles of predicted probabilities across nine homologous tissue datasets (brain, liver, and kidney across human, mouse, and rat). Across all nine datasets, the predicted probabilities follow a distinct bimodal distribution concentrated near 0 and 1, with relatively low density near the 0.5 decision boundary. This distribution indicates that HMB-Net assigns high-confidence probability estimates to most test sequences rather than producing ambiguous intermediate scores.

#### 3.5.3. Motif Saliency and Gradient Attribution Analysis

**Figure 9:**
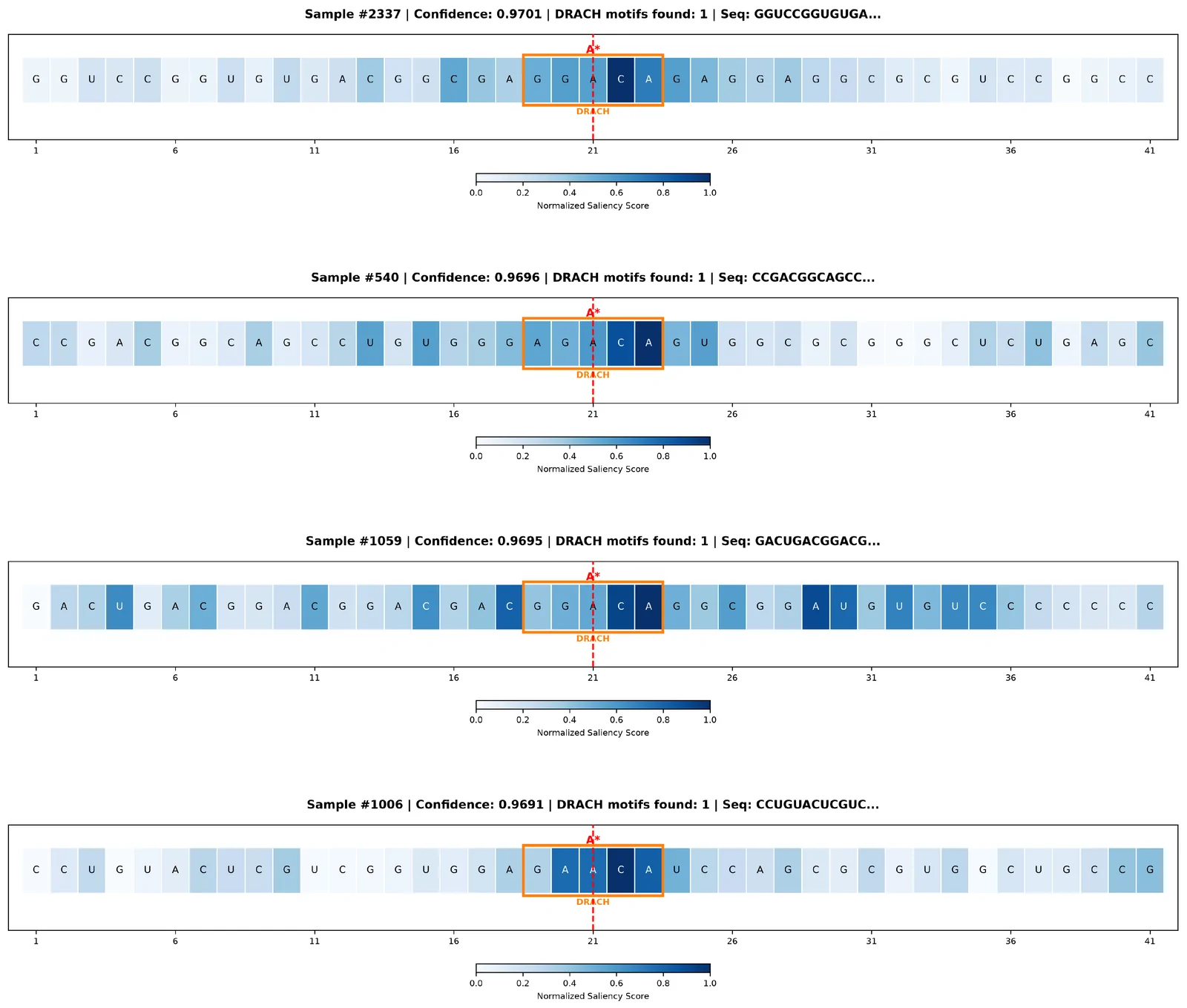
Base-resolution saliency attribution heatmaps across the 41-nt core sequence window (positions 1–41 centered on A* at position 21; trailing zero-padded positions 42–101 are omitted) on the top five high-confidence true-positive human liver (h_l) independent test sequences containing a central DRACH motif. High attribution scores (dark blue) concentrate on the immediate flanking positions (19–23) of the canonical DRACH/RRACH consensus motif. The vertical red dashed line and asterisk (A*) mark the central candidate modification site at position 21, and the orange dashed boxes highlight the canonical DRACH/RRACH consensus motifs at positions 19–23.

To examine which nucleotide positions drive the predictions of HMB-Net, we computed base-resolution attribution scores using Integrated Gradients [34]. Figure 9 displays the active 41-nt core sequence window (positions 1–41 centered at position 21, after trimming the zero-padded positions 42–101 of the 101-nt input buffer) for the top five high-confidence true-positive sequences containing a central DRACH motif from the human liver (h_l) independent test set (Samples #2337, #540, #1059, #1006, and #2484, selected by ranking true-positive test predictions in descending order of predicted probability). Across all five sequences, the highest attribution weights cluster around the immediate flanking positions (19–23) of the central DRACH/RRACH consensus motif (GGACA, AGACA, and GAACA). Because the central candidate nucleotide (A* at position 21) is shared across positive and negative windows, the network relies primarily on the surrounding motif positions to determine modification status; similarly, attribution analyses on human m^5^C (Atlas_h_m5C) and m^1^A (Atlas_h_m1A) concentrate on GC-rich (CCCGG) and purine-rich (GAACC) central motifs, respectively. This pattern confirms that HMB-Net learns biologically relevant sequence determinants consistent with methyltransferase recognition preferences.

## 4. Discussion

Accurate transcriptome-wide identification of RNA modification sites requires capturing both distal sequence context and short, position-specific consensus motifs. In this study, we developed HMB-Net to address the trade-off between global context modeling and local motif sensitivity through a cascaded Mamba-BiLSTM architecture.

### 4.1. Effectiveness of the “Latent-to-Motif” Hierarchy

A central design choice of HMB-Net is its sequential “Latent-to-Motif” hierarchy. As shown in our module-ordering ablation study (Section 3.4.2), both reversing the encoding order (R-HMB-Net, where the Bi-LSTM precedes the Mamba block) and placing the two modules in a parallel dual-branch configuration (P-HMB-Net) lead to consistent performance degradation in both ROC-AUC and MCC across the benchmark datasets under the same parameter budget. When a localized recurrent network processes raw nucleotide embeddings first or in parallel without global conditioning, it can overfit local sequence fluctuations, which subsequently degrade the downstream or fused representations. By contrast, placing the Selective SSM (Mamba) block first establishes a denoised, long-range contextual representation across the sequence window, enabling the downstream Bi-LSTM and bidirectional cross-attention modules to refine local motif boundaries more effectively.

### 4.2. Predictive Calibration and Cross-Modification Generalization

Beyond thresholded classification metrics, the probability density profiles (Section 3.5.2) show that HMB-Net produces well-calibrated bimodal score distributions concentrated near 0 and 1, with few ambiguous predictions in the intermediate range (0.4 to 0.6). Furthermore, evaluation across the 9 RMBase and m6A-Atlas benchmarks (Section 3.3) demonstrates that the architecture generalizes effectively across m^6^A, m^5^C, and m^1^A modifications in human, mouse, and rat, maintaining balanced sensitivity and specificity across diverse biochemical modification types while also retaining strong zero-shot cross-species transferability across homologous mammalian tissues.

### 4.3. Biological Interpretability and Future Directions

For sequence-based predictors to be useful in biological discovery, their predictions should reflect genuine biochemical signals rather than dataset artifacts. The PCA and t-SNE projections (Section 3.5.1) show that HMB-Net maps raw nucleotide windows into well-separated clusters corresponding to methylated and non-methylated states. Moreover, base-resolution integrated gradient maps (Section 3.5.3) demonstrate that the model assigns high importance to the flanking bases of canonical modification motifs (such as DRACH/RRACH for m^6^A, CCCGG for m^5^C, and GAACC for m^1^A) without prior motif supervision. Despite these strengths, several limitations should be noted. First, while our benchmark datasets use a balanced 1:1 positive-to-negative ratio following standard evaluation protocols, unmodified candidate sites substantially outnumber modified sites in realistic transcriptome-wide scanning; practical genome-wide applications therefore require probability recalibration, Precision–Recall evaluation, or more stringent decision thresholds (e.g., $\hat{y}>0.90$) to control false-positive calls. Second, HMB-Net operates on fixed 101-nt input windows (41-nt core sequence windows) using primary sequence alone and trains separate models for each modification type, which cannot capture distal cis-regulatory elements or long-range secondary structures beyond the local window. Third, although all benchmark datasets were curated from experimentally verified high-throughput sequencing repositories, novel candidate sites predicted by HMB-Net have not yet been prospectively validated by independent wet-lab assays; future work could combine wet-lab validation with predicted RNA secondary structure features, isoform-level transcript coordinates, multi-task joint modification heads, or direct RNA nanopore current signals to further enhance site-level modification calling in complex cellular environments.

## 5. Conclusions

In this study, we presented HMB-Net, an interpretable hybrid deep learning framework that integrates the linear-time global sequence modeling of Selective State Space Models (Mamba) with the local motif sensitivity of multi-layer Bi-LSTMs. Through its sequential “Latent-to-Motif” encoding hierarchy and bidirectional cross-attention fusion, HMB-Net effectively balances long-range contextual representation with fine-grained local motif extraction. Benchmarking across 20 non-redundant mammalian datasets covering m^6^A, m^5^C, and m^1^A modifications demonstrates that HMB-Net consistently outperforms representative CNN-, RNN-, and Transformer-based baselines. Furthermore, latent space visualizations and gradient attribution analyses confirm that the model autonomously recovers canonical consensus motifs such as RRACH, providing an accurate and interpretable computational tool for epitranscriptomic analysis.

## Figures and Tables

**Table 1:** Statistics of the constructed benchmark datasets.

Database	Type	Species	Tissue	Positive Training	Positive Testing	Negative Training	Negative Testing
Dao et al. [31]	m^6^A	Human	Brain	4605	4604	4605	4604
			Liver	2634	2634	2634	2634
			Kidney	4574	4573	4574	4573
		Mouse	Brain	8025	8025	8025	8025
			Liver	4133	4133	4133	4133
			Kidney	3953	3952	3953	3952
			Heart	2201	2200	2201	2200
			Testis	4704	4706	4704	4706
		Rat	Brain	2352	2351	2352	2351
			Liver	1762	1762	1762	1762
			Kidney	3433	3432	3433	3432
RMBase [32]	m^6^A	Human	N/A	4605	4573	4605	4573
		Mouse	N/A	4707	4706	4707	4706
		Rat	N/A	3433	3432	3433	3432
m6A-Atlas [33]	m^6^A	Human	N/A	3397	4573	3397	4573
		Mouse	N/A	3953	3952	3953	3952
		Rat	N/A	3174	3173	3174	3173
	m^5^C	Human	N/A	1787	1787	1787	1787
	m^1^A	Human	N/A	1924	1785	1924	1785
		Mouse	N/A	2233	2233	2233	2233

**Table 2:** Performance comparison of HMB-Net and baseline methods on 11 tissue-specific m^6^A datasets.

Benchmark	Model	ACC	Sn	Sp	Pre	F1	MCC	ROC
h_b	DeepMRMP	0.7183	0.7416	0.6949	0.7085	0.7247	0.4370	0.7974
	DeepPromise	0.7204	0.7492	0.6916	0.7084	0.7282	0.4415	0.7833
	M6A-BERT	0.7232	0.7557	0.6906	0.7095	0.7319	0.4472	0.8015
	**HMB-Net**	**0.7433**	**0.7906**	**0.6959**	**0.7222**	**0.7549**	**0.4887**	**0.8229**
h_k	DeepMRMP	0.7644	0.7519	0.7768	0.7711	0.7614	0.5289	0.8370
	DeepPromise	0.7561	0.7213	0.7908	0.7752	0.7473	0.5133	0.8316
	M6A-BERT	0.7760	0.7464	0.8055	0.7933	0.7691	0.5529	0.8630
	**HMB-Net**	**0.7897**	**0.7538**	**0.8255**	**0.8120**	**0.7818**	**0.5808**	**0.8807**
h_l	DeepMRMP	0.8084	0.8065	0.8102	0.8095	0.8080	0.6167	0.8817
	DeepPromise	0.7923	0.7913	0.7932	0.7928	0.7920	0.5845	0.8713
	M6A-BERT	0.7987	0.8027	0.7947	0.7963	0.7995	0.5974	0.8810
	**HMB-Net**	**0.8144**	**0.8159**	**0.8128**	**0.8134**	**0.8146**	**0.6287**	**0.8905**
m_b	DeepMRMP	0.7779	0.7919	0.7639	0.7703	0.7810	0.5560	0.8671
	DeepPromise	0.7629	0.7651	0.7607	0.7617	0.7634	0.5258	0.8416
	M6A-BERT	0.7633	0.7695	0.7570	0.7600	0.7647	0.5265	0.8456
	**HMB-Net**	**0.7961**	**0.8264**	**0.7657**	**0.7791**	**0.8021**	**0.5932**	**0.8825**
m_h	DeepMRMP	0.7307	0.7659	0.6955	0.7155	0.7398	0.4625	0.8162
	DeepPromise	0.7254	0.7568	0.6939	0.7120	0.7337	0.4516	0.7949
	M6A-BERT	0.7319	0.7682	0.6955	0.7161	0.7412	0.4649	0.8156
	**HMB-Net**	**0.7507**	**0.8041**	**0.6973**	**0.7265**	**0.7633**	**0.5043**	**0.8331**
m_k	DeepMRMP	0.8020	0.8063	0.7977	0.7994	0.8028	0.6040	0.8824
	DeepPromise	0.7856	0.7863	0.7848	0.7851	0.7857	0.5711	0.8704
	M6A-BERT	0.7914	0.7939	0.7889	0.7900	0.7919	0.5828	0.8608
	**HMB-Net**	**0.8137**	**0.8229**	**0.8044**	**0.8080**	**0.8154**	**0.6274**	**0.8942**
m_l	DeepMRMP	0.6998	0.7481	0.6515	0.6822	0.7136	0.4015	0.7624
	DeepPromise	0.6857	0.7205	0.6508	0.6736	0.6963	0.3722	0.7488
	M6A-BERT	0.6891	0.7276	0.6505	0.6755	0.7006	0.3792	0.7484
	**HMB-Net**	**0.7296**	**0.8076**	**0.6516**	**0.6986**	**0.7492**	**0.4649**	**0.8100**
m_t	DeepMRMP	0.7456	0.7566	0.7346	0.7403	0.7484	0.4913	0.8302
	DeepPromise	0.7455	0.7566	0.7343	0.7401	0.7483	0.4910	0.8340
	M6A-BERT	0.7476	0.7609	0.7343	0.7412	0.7509	0.4954	0.8369
	**HMB-Net**	**0.7790**	**0.8230**	**0.7350**	**0.7564**	**0.7883**	**0.5602**	**0.8604**
r_b	DeepMRMP	0.7673	0.7728	0.7617	0.7643	0.7685	0.5345	0.8500
	DeepPromise	0.7577	0.7596	0.7558	0.7567	0.7581	0.5154	0.8340
	M6A-BERT	0.7599	0.7596	0.7601	0.7600	0.7598	0.5197	0.8459
	**HMB-Net**	**0.7827**	**0.8035**	**0.7618**	**0.7713**	**0.7871**	**0.5658**	**0.8663**
r_k	DeepMRMP	0.8239	0.8399	0.8079	0.8139	0.8267	0.6481	0.9065
	DeepPromise	0.8070	0.8076	0.8064	0.8066	0.8071	0.6140	0.8904
	M6A-BERT	0.8172	0.8268	0.8076	0.8112	0.8189	0.6345	0.8912
	**HMB-Net**	**0.8369**	**0.8648**	**0.8089**	**0.8190**	**0.8413**	**0.6748**	**0.9143**
r_l	DeepMRMP	0.7873	0.7898	0.7847	0.7858	0.7878	0.5745	0.8643
	DeepPromise	0.7816	0.7819	0.7812	0.7814	0.7816	0.5631	0.8732
	M6A-BERT	0.7983	0.8125	0.7841	0.7901	0.8011	0.5968	0.8954
	**HMB-Net**	**0.8173**	**0.8485**	**0.7860**	**0.7986**	**0.8228**	**0.6357**	**0.9004**

Note: Bold values indicate the best performance on each benchmark dataset.

## Data Availability

The source code and benchmark datasets used in this study are publicly available at https://github.com/raozeng/HMB-Net (accessed on 3 November 2026).

## Supplementary Materials

The following supporting information can be downloaded at: https://www.mdpi.com/article/10.3390/biology15212028/s1, Table S1: Performance comparison of HMB-Net and baseline methods on cross-modification datasets; Table S2: Results of ablation study and architectural analysis across all 20 benchmark datasets.

## References

[1] Boccaletto P., Machnicka M.A., Purta E., Piątkowski P., Bagiński B., Wirecki T.K., de Crécy-Lagard V., Ross R., Limbach P.A., Kotter A. (2018). MODOMICS: A database of RNA modification pathways. 2017 update. Nucleic Acids Res..

[2] Wiener D., Schwartz S. (2021). The epitranscriptome beyond m6A. Nat. Rev. Genet..

[3] Zhao B.S., Roundtree I.A., He C. (2017). Post-transcriptional gene regulation by mRNA modifications. Nat. Rev. Mol. Cell Biol..

[4] Roundtree I.A., Evans M.E., Pan T., He C. (2017). Dynamic RNA Modifications in Gene Expression Regulation. Cell.

[5] Yang Y., Hsu P.J., Chen Y.S., Yang Y.G. (2018). Dynamic transcriptomic m6A decoration: Writers, erasers, readers and functions in RNA metabolism. Cell Res..

[6] Liu W.W., Zheng S.Q., Li T., Fei Y.F., Wang C., Zhang S., Wang F., Jiang G.M., Wang H. (2024). RNA modifications in cellular metabolism: Implications for metabolism-targeted therapy and immunotherapy. Signal Transduct. Target. Ther..

[7] Barbieri I., Kouzarides T. (2020). Role of RNA modifications in cancer. Nat. Rev. Cancer.

[8] Dominissini D., Moshitch-Moshkovitz S., Schwartz S., Salmon-Divon M., Ungar L., Osenberg S., Cesarkas K., Jacob-Hirsch J., Amariglio N., Kupiec M. (2012). Topology of the human and mouse m6A RNA methylomes revealed by m6A-seq. Nature.

[9] Meyer K.D., Saletore Y., Zumbo P., Elemento O., Mason C.E., Jaffrey S.R. (2012). Comprehensive Analysis of mRNA Methylation Reveals Enrichment in 3’ UTRs and near Stop Codons. Cell.

[10] Linder B., Grozhik A.V., Olarerin-George A.O., Meydan C., Mason C.E., Jaffrey S.R. (2015). Single-nucleotide-resolution mapping of m6A and m6Am throughout the transcriptome. Nat. Methods.

[11] Park D., Cenik C. (2024). Long-read RNA sequencing reveals allele-specific N^6^-methyladenosine modifications. bioRxiv.

[12] Angermueller C., Pärnamaa T., Parts L., Stegle O. (2016). Deep learning for computational biology. Mol. Syst. Biol..

[13] Chen Z., Zhao P., Li F., Wang Y., Smith A.I., Webb G.I., Akutsu T., Baggag A., Bensmail H., Song J. (2020). Comprehensive review and assessment of computational methods for predicting RNA post-transcriptional modification sites from RNA sequences. Brief Bioinform..

[14] Chen W., Feng P., Ding H., Lin H., Chou K.C. (2015). IRNA-Methyl: Identifying N6-methyladenosine sites using pseudo nucleotide composition. Anal. Biochem..

[15] Chen W., Feng P., Yang H., Ding H., Lin H., Chou K.C. (2017). IRNA-AI: Identifying the adenosine to inosine editing sites in RNA sequences. Oncotarget.

[16] Song J., Zhai J., Bian E., Song Y., Yu J., Ma C. (2018). Transcriptome-Wide Annotation of m5C RNA Modifications Using Machine Learning. Front. Plant Sci..

[17] Bi Y., Xiang D., Ge Z., Li F., Jia C., Song J. (2020). An Interpretable Prediction Model for Identifying N7-Methylguanosine Sites Based on XGBoost and SHAP. Mol. Ther. Nucleic Acids.

[18] Lundberg S.M., Lee S.I. A unified approach to interpreting model predictions. Proceedings of the Advances in Neural Information Processing Systems (NeurIPS).

[19] Zhang Y., Hamada M. (2018). DeepM6ASeq: Prediction and characterization of m6A-containing sequences using deep learning. BMC Bioinform..

[20] Kim Y. Convolutional Neural Networks for Sentence Classification. Proceedings of the 2014 Conference on Empirical Methods in Natural Language Processing (EMNLP).

[21] Hochreiter S., Schmidhuber J. (1997). Long Short-Term Memory. Neural Comput..

[22] Zhou P., Shi W., Tian J., Qi Z., Li B., Hao H., Xu B. Attention-Based Bidirectional Long Short-Term Memory Networks for Relation Classification. Proceedings of the 54th Annual Meeting of the Association for Computational Linguistics (Volume 2: Short Papers).

[23] Sun P., Chen Y., Liu B., Gao Y., Han Y., He F., Ji J. (2019). DeepMRMP: A new predictor for multiple types of RNA modification sites using deep learning. Math. Biosci. Eng..

[24] Vaswani A., Shazeer N., Parmar N., Uszkoreit J., Jones L., Gomez A.N., Kaiser L., Polosukhin I. Attention is all you need. Proceedings of the Advances in Neural Information Processing Systems (NeurIPS).

[25] Dalla-Torre H., Gonzalez L., Mendoza-Revilla J., Lopez Carranza N., Grzywaczewski A.H., Oteri F., Dallago C., Trop E., De Almeida B.P., Sirelkhatim H. (2025). Nucleotide Transformer: Building and evaluating robust foundation models for human genomics. Nat. Methods.

[26] Zhang T.H., Jo S., Zhang M., Wang K., Gao S.J., Huang Y. (2024). Understanding YTHDF2-mediated mRNA degradation by m6A-BERT-Deg. Brief. Bioinform..

[27] Choromanski K., Likhosherstov V., Dohan D., Song X., Gane A., Sarlos T., Hawkins P., Davis J., Mohiuddin A., Kaiser L. Rethinking attention with performers. Proceedings of the International Conference on Learning Representations (ICLR).

[28] Gu A., Dao T. (2023). Mamba: Linear-time sequence modeling with selective state spaces. arXiv.

[29] Gu A., Goel K., Ré C. Efficiently modeling long sequences with structured state spaces. Proceedings of the International Conference on Learning Representations (ICLR).

[30] Fu L., Niu B., Zhu Z., Wu S., Li W. (2012). CD-HIT: Accelerated for clustering the next-generation sequencing data. Bioinformatics.

[31] Dao F.Y., Lv H., Yang Y.H., Zulfiqar H., Gao H., Lin H. (2020). Computational identification of N6-methyladenosine sites in multiple tissues of mammals. Comput. Struct. Biotechnol. J..

[32] Xuan J.J., Sun W.J., Lin P.H., Zhou K.R., Liu S., Zheng L.L., Qu L.H., Yang J.H. (2018). RMBase v2.0: Deciphering the map of RNA modifications from epitranscriptome sequencing data. Nucleic Acids Res..

[33] Tang Y., Chen K., Song B., Ma J., Wu X., Xu Q., Wei Z., Su J., Liu G., Rong R. (2021). M6A-Atlas: A comprehensive knowledgebase for unraveling the *N*6-methyladenosine (m6A) epitranscriptome. Nucleic Acids Res..

[34] Sundararajan M., Taly A., Yan Q. Axiomatic attribution for deep networks. Proceedings of the 34th International Conference on Machine Learning (ICML).

