# Supplementary material for: HMB-Net: Synergizing Latent Sequential Dynamics and Local Sequence Motifs for Robust RNA Methylation Prediction
Source: Biology (Basel). 2026 Nov 7;15(21):2028. doi: 10.3390/biology15212028 (PMC13761918; doi:10.3390/biology15212028)
Supplement: Supplementary file 1 [file biology-15-02028-s001.zip › biology-4672210-supplementary.pdf]

biology

MDPI

Article

HMB-Net: Synergizing Latent Sequential Dynamics and Local Sequence Motifs for Robust RNA Methylation Prediction

Fang Fang [1], Dong Xu [2,*] and Rao Zeng [2,3,*]

[1] School of Information Engineering, Hainan Vocational University of Science and Technology, Haikou 571126, China; fangf.0110@gmail.com
[2] School of Artificial Intelligence, Hainan Normal University, Haikou 571158, China
[3] Key Laboratory of Data Science and Smart Education, Ministry of Education, Hainan Normal University, Haikou 571158, China
* Correspondence: xdong@hainnu.edu.cn (D.X.); rao.zeng@hainnu.edu.cn (R.Z.)

1. Supplementary Materials

The detailed numerical metrics corresponding to the cross-modification radar charts in Section 3.3 are provided in Table S1.

Table S1. Performance comparison of HMB-Net and baseline methods on cross-modification datasets.

Benchmark	Model	ACC	Sn	Sp	Pre	F1	MCC	ROC
Atlas_h_m1A	DeepMRMP	0.9688	0.9636	0.9740	0.9737	0.9686	0.9377	0.9928
	DeepPromise	0.9519	0.9505	0.9532	0.9531	0.9518	0.9037	0.9852
	M6A-BERT	0.9571	0.9583	0.9558	0.9559	0.9571	0.9141	0.9886
	**HMB-Net**	**0.9773**	**0.9672**	**0.9873**	**0.9870**	**0.9770**	**0.9547**	**0.9954**
Atlas_h_m5C	DeepMRMP	0.9008	0.8988	0.9027	0.9023	0.9005	0.8015	0.9658
	DeepPromise	0.8911	0.8911	0.8911	0.8911	0.8911	0.7822	0.9589
	M6A-BERT	0.8932	0.8953	0.8911	0.8916	0.8934	0.7864	0.9646
	**HMB-Net**	**0.9259**	**0.9014**	**0.9503**	**0.9477**	**0.9240**	**0.8527**	**0.9779**
Atlas_h_m6A	DeepMRMP	0.6662	0.6819	0.6504	0.6611	0.6713	0.3325	0.7305
	DeepPromise	0.6987	0.7492	0.6482	0.6805	0.7132	0.3994	0.7779
	M6A-BERT	0.6986	0.7500	0.6472	0.6801	0.7133	0.3993	0.7421
	**HMB-Net**	**0.8069**	**0.9613**	**0.6525**	**0.7345**	**0.8327**	**0.6453**	**0.9435**
Atlas_m_m1A	DeepMRMP	0.8309	0.8047	0.8571	0.8492	0.8264	0.6627	0.9098
	DeepPromise	0.8118	0.8000	0.8235	0.8193	0.8095	0.6237	0.8984
	M6A-BERT	0.7954	0.7865	0.8043	0.8008	0.7936	0.5909	0.8773
	**HMB-Net**	**0.8488**	**0.8172**	**0.8803**	**0.8722**	**0.8438**	**0.6989**	**0.9429**
Atlas_m_m6A	DeepMRMP	0.8818	0.8622	0.9013	0.8973	0.8794	0.7641	0.9582
	DeepPromise	0.8615	0.8622	0.8608	0.8610	0.8616	0.7230	0.9421
	M6A-BERT	0.8711	0.8609	0.8812	0.8787	0.8697	0.7423	0.9490
	**HMB-Net**	**0.8931**	**0.8682**	**0.9180**	**0.9137**	**0.8904**	**0.7872**	**0.9684**
Atlas_r_m6A	DeepMRMP	0.8881	0.9118	0.8644	0.8705	0.8907	0.7771	0.9517
	DeepPromise	0.8685	0.8787	0.8583	0.8611	0.8698	0.7372	0.9397
	M6A-BERT	0.8574	0.8596	0.8551	0.8557	0.8576	0.7147	0.9375
	**HMB-Net**	**0.8971**	**0.9297**	**0.8644**	**0.8727**	**0.9003**	**0.7958**	**0.9616**

Continued on next page

Check for updates

Academic Editor: Yuan Zhou

Received: 19 October 2026
Revised: 3 November 2026
Accepted: 5 November 2026
Published: 7 November 2026

Copyright: © 2026 by the authors. Licensee MDPI, Basel, Switzerland. This article is an open access article distributed under the terms and conditions of the Creative Commons Attribution (CC BY) license.

Table S1. Performance comparison of HMB-Net and baseline methods on cross-modification datasets. (continued)

Benchmark	Model	ACC	Sn	Sp	Pre	F1	MCC	ROC
RMBase_h_m6A	DeepMRMP	0.8719	0.8458	0.8979	0.8923	0.8684	0.7447	0.9537
	DeepPromise	0.8410	0.8317	0.8502	0.8474	0.8395	0.6820	0.9243
	M6A-BERT	0.8573	0.8263	0.8882	0.8808	0.8527	0.7159	0.9414
	**HMB-Net**	**0.8880**	**0.8606**	**0.9153**	**0.9104**	**0.8848**	**0.7771**	**0.9689**
RMBase_m_m6A	DeepMRMP	0.8439	0.8937	0.7941	0.8128	0.8513	0.6912	0.9458
	DeepPromise	0.8332	0.8746	0.7917	0.8076	0.8398	0.6686	0.9394
	M6A-BERT	0.8423	0.8907	0.7938	0.8120	0.8495	0.6877	0.9419
	**HMB-Net**	**0.8587**	**0.9199**	**0.7974**	**0.8195**	**0.8668**	**0.7227**	**0.9573**
RMBase_r_m6A	DeepMRMP	0.9185	0.9185	0.9184	0.9184	0.9184	0.8369	0.9799
	DeepPromise	0.9068	0.9083	0.9052	0.9055	0.9069	0.8135	0.9776
	M6A-BERT	0.9075	0.9054	0.9096	0.9092	0.9073	0.8150	0.9759
	**HMB-Net**	**0.9264**	**0.9318**	**0.9210**	**0.9218**	**0.9268**	**0.8528**	**0.9844**

The detailed numerical results for the ablation study and module-ordering analysis presented in Section 3.4 are provided in Table S2.

Table S2. Results of ablation study and architectural analysis across all 20 benchmark datasets.

Benchmark	Model	ACC	Sn	Sp	Pre	F1	MCC	ROC
h_b	BiLSTMOnly	0.6846	0.6992	0.6699	0.6793	0.6891	0.3693	0.7551
	MambaOnly	0.7243	0.7579	0.6906	0.7101	0.7332	0.4495	0.8035
	R-HMB-Net	0.7291	0.7644	0.6938	0.7140	0.7383	0.4593	0.8044
	P-HMB-Net	0.7346	0.7744	0.6947	0.7172	0.7447	0.4706	0.8118
	**HMB-Net**	**0.7433**	**0.7906**	**0.6959**	**0.7222**	**0.7549**	**0.4887**	**0.8229**
h_k	BiLSTMOnly	0.7549	0.7169	0.7929	0.7759	0.7452	0.5113	0.8333
	MambaOnly	0.7524	0.7027	0.8021	0.7803	0.7395	0.5073	0.8378
	R-HMB-Net	0.7775	0.7473	0.8077	0.7953	0.7706	0.5560	0.8680
	P-HMB-Net	0.7818	0.7493	0.8142	0.8013	0.7744	0.5647	0.8731
	**HMB-Net**	**0.7897**	**0.7538**	**0.8255**	**0.8120**	**0.7818**	**0.5808**	**0.8807**
h_l	BiLSTMOnly	0.7799	0.7799	0.7799	0.7799	0.7799	0.5598	0.8599
	MambaOnly	0.7847	0.7837	0.7856	0.7852	0.7844	0.5693	0.8669
	R-HMB-Net	0.7989	0.7951	0.8027	0.8012	0.7981	0.5978	0.8827
	P-HMB-Net	0.8047	0.8030	0.8064	0.8057	0.8043	0.6094	0.8858
	**HMB-Net**	**0.8144**	**0.8159**	**0.8128**	**0.8134**	**0.8146**	**0.6287**	**0.8905**
m_b	BiLSTMOnly	0.7583	0.7607	0.7558	0.7570	0.7588	0.5165	0.8362
	MambaOnly	0.7751	0.7944	0.7558	0.7649	0.7794	0.5506	0.8538
	R-HMB-Net	0.7813	0.8019	0.7607	0.7702	0.7857	0.5631	0.8694
	P-HMB-Net	0.7869	0.8112	0.7625	0.7735	0.7919	0.5744	0.8746
	**HMB-Net**	**0.7961**	**0.8264**	**0.7657**	**0.7791**	**0.8021**	**0.5932**	**0.8825**
m_h	BiLSTMOnly	0.7094	0.7347	0.6841	0.6993	0.7166	0.4193	0.7903
	MambaOnly	0.7182	0.7500	0.6864	0.7052	0.7269	0.4373	0.7916
	R-HMB-Net	0.7296	0.7659	0.6932	0.7140	0.7390	0.4603	0.8141
	P-HMB-Net	0.7377	0.7806	0.6947	0.7189	0.7485	0.4771	0.8217
	**HMB-Net**	**0.7507**	**0.8041**	**0.6973**	**0.7265**	**0.7633**	**0.5043**	**0.8331**

Continued on next page

Table S2. Results of ablation study and architectural analysis across all 20 benchmark datasets. (continued)

Benchmark	Model	ACC	Sn	Sp	Pre	F1	MCC	ROC
m_k	BiLSTMOnly	0.7787	0.7747	0.7826	0.7809	0.7778	0.5573	0.8609
	MambaOnly	0.7869	0.7911	0.7826	0.7844	0.7877	0.5737	0.8710
	R-HMB-Net	0.7989	0.8025	0.7952	0.7967	0.7996	0.5977	0.8821
	P-HMB-Net	0.8044	0.8102	0.7986	0.8009	0.8055	0.6088	0.8869
	**HMB-Net**	**0.8137**	**0.8229**	**0.8044**	**0.8080**	**0.8154**	**0.6274**	**0.8942**
m_l	BiLSTMOnly	0.6640	0.7052	0.6227	0.6515	0.6773	0.3290	0.7372
	MambaOnly	0.6049	0.5828	0.6270	0.6098	0.5960	0.2100	0.6485
	R-HMB-Net	0.7094	0.7687	0.6500	0.6871	0.7256	0.4217	0.7748
	P-HMB-Net	0.7171	0.7835	0.6506	0.6916	0.7347	0.4380	0.7889
	**HMB-Net**	**0.7296**	**0.8076**	**0.6516**	**0.6986**	**0.7492**	**0.4649**	**0.8100**
m_t	BiLSTMOnly	0.7423	0.7524	0.7322	0.7375	0.7449	0.4847	0.8245
	MambaOnly	0.7461	0.7588	0.7333	0.7399	0.7492	0.4923	0.8288
	R-HMB-Net	0.7531	0.7728	0.7333	0.7434	0.7578	0.5065	0.8423
	P-HMB-Net	0.7629	0.7918	0.7340	0.7485	0.7695	0.5267	0.8495
	**HMB-Net**	**0.7790**	**0.8230**	**0.7350**	**0.7564**	**0.7883**	**0.5602**	**0.8604**
r_b	BiLSTMOnly	0.7514	0.7516	0.7511	0.7512	0.7514	0.5027	0.8341
	MambaOnly	0.7532	0.7532	0.7532	0.7532	0.7532	0.5064	0.8267
	R-HMB-Net	0.7588	0.7601	0.7574	0.7581	0.7591	0.5175	0.8385
	P-HMB-Net	0.7679	0.7768	0.7589	0.7631	0.7699	0.5358	0.8496
	**HMB-Net**	**0.7827**	**0.8035**	**0.7618**	**0.7713**	**0.7871**	**0.5658**	**0.8663**
r_k	BiLSTMOnly	0.8041	0.8047	0.8035	0.8037	0.8042	0.6082	0.8771
	MambaOnly	0.8055	0.8061	0.8049	0.8051	0.8056	0.6110	0.8857
	R-HMB-Net	0.8194	0.8324	0.8064	0.8113	0.8217	0.6390	0.8996
	P-HMB-Net	0.8261	0.8448	0.8074	0.8143	0.8293	0.6527	0.9055
	**HMB-Net**	**0.8369**	**0.8648**	**0.8089**	**0.8190**	**0.8413**	**0.6748**	**0.9143**
r_l	BiLSTMOnly	0.7816	0.7790	0.7841	0.7830	0.7810	0.5631	0.8663
	MambaOnly	0.7858	0.7926	0.7790	0.7820	0.7873	0.5717	0.8652
	R-HMB-Net	0.7801	0.7790	0.7812	0.7807	0.7798	0.5602	0.8663
	P-HMB-Net	0.7943	0.8053	0.7832	0.7879	0.7965	0.5886	0.8799
	**HMB-Net**	**0.8173**	**0.8485**	**0.7860**	**0.7986**	**0.8228**	**0.6357**	**0.9004**
Atlas_h_m1A	BiLSTMOnly	0.9364	0.9403	0.9325	0.9330	0.9366	0.8728	0.9801
	MambaOnly	0.9468	0.9403	0.9532	0.9526	0.9464	0.8936	0.9855
	R-HMB-Net	0.9610	0.9583	0.9636	0.9634	0.9608	0.9219	0.9901
	P-HMB-Net	0.9672	0.9616	0.9728	0.9725	0.9670	0.9345	0.9922
	**HMB-Net**	**0.9773**	**0.9672**	**0.9873**	**0.9870**	**0.9770**	**0.9547**	**0.9954**
Atlas_h_m5C	BiLSTMOnly	0.8913	0.8953	0.8872	0.8881	0.8917	0.7825	0.9645
	MambaOnly	0.8952	0.8915	0.8988	0.8981	0.8948	0.7903	0.9638
	R-HMB-Net	0.8952	0.8992	0.8911	0.8920	0.8956	0.7903	0.9632
	P-HMB-Net	0.9068	0.8998	0.9138	0.9126	0.9062	0.8137	0.9694
	**HMB-Net**	**0.9259**	**0.9014**	**0.9503**	**0.9477**	**0.9240**	**0.8527**	**0.9779**
Atlas_h_m6A	BiLSTMOnly	0.6922	0.7459	0.6384	0.6735	0.7079	0.3865	0.7687
	MambaOnly	0.7085	0.7698	0.6471	0.6857	0.7253	0.4201	0.7779
	R-HMB-Net	0.7079	0.7700	0.6457	0.6849	0.7250	0.4189	0.7764
	P-HMB-Net	0.7455	0.8427	0.6483	0.7055	0.7680	0.5005	0.8441
	**HMB-Net**	**0.8069**	**0.9613**	**0.6525**	**0.7345**	**0.8327**	**0.6453**	**0.9435**

Continued on next page

Table S2. Results of ablation study and architectural analysis across all 20 benchmark datasets. (continued)

Benchmark	Model	ACC	Sn	Sp	Pre	F1	MCC	ROC
Atlas_m_m1A	BiLSTMOnly	0.8001	0.7844	0.8157	0.8097	0.7968	0.6004	0.8824
	MambaOnly	0.8027	0.7818	0.8235	0.8158	0.7984	0.6058	0.8940
	R-HMB-Net	0.8260	0.7812	0.8708	0.8581	0.8178	0.6546	0.9031
	P-HMB-Net	0.8344	0.7952	0.8735	0.8628	0.8276	0.6708	0.9190
	**HMB-Net**	**0.8488**	**0.8172**	**0.8803**	**0.8722**	**0.8438**	**0.6989**	**0.9429**
Atlas_m_m6A	BiLSTMOnly	0.8596	0.8534	0.8658	0.8641	0.8587	0.7193	0.9429
	MambaOnly	0.8672	0.8571	0.8772	0.8747	0.8658	0.7344	0.9501
	R-HMB-Net	0.8742	0.8622	0.8861	0.8833	0.8726	0.7485	0.9529
	P-HMB-Net	0.8814	0.8645	0.8983	0.8947	0.8793	0.7632	0.9591
	**HMB-Net**	**0.8931**	**0.8682**	**0.9180**	**0.9137**	**0.8904**	**0.7872**	**0.9684**
Atlas_r_m6A	BiLSTMOnly	0.8613	0.8675	0.8551	0.8569	0.8622	0.7227	0.9377
	MambaOnly	0.8582	0.8644	0.8520	0.8538	0.8591	0.7165	0.9368
	R-HMB-Net	0.8818	0.9024	0.8612	0.8667	0.8842	0.7642	0.9476
	P-HMB-Net	0.8882	0.9134	0.8630	0.8696	0.8910	0.7774	0.9532
	**HMB-Net**	**0.8971**	**0.9297**	**0.8644**	**0.8727**	**0.9003**	**0.7958**	**0.9616**
RMBase_h_m6A	BiLSTMOnly	0.8475	0.8046	0.8903	0.8800	0.8406	0.6975	0.9328
	MambaOnly	0.8855	0.8599	0.9110	0.9062	0.8824	0.7719	0.9641
	R-HMB-Net	0.8860	0.8578	0.9142	0.9091	0.8827	0.7732	0.9603
	P-HMB-Net	0.8868	0.8589	0.9146	0.9096	0.8835	0.7747	0.9660
	**HMB-Net**	**0.8880**	**0.8606**	**0.9153**	**0.9104**	**0.8848**	**0.7771**	**0.9689**
RMBase_m_m6A	BiLSTMOnly	0.8157	0.8482	0.7832	0.7964	0.8215	0.6327	0.9262
	MambaOnly	0.8258	0.8747	0.7768	0.7967	0.8339	0.6546	0.9300
	R-HMB-Net	0.8433	0.8927	0.7938	0.8124	0.8507	0.6899	0.9441
	P-HMB-Net	0.8491	0.9030	0.7952	0.8151	0.8568	0.7023	0.9494
	**HMB-Net**	**0.8587**	**0.9199**	**0.7974**	**0.8195**	**0.8668**	**0.7227**	**0.9573**
RMBase_r_m6A	BiLSTMOnly	0.9010	0.9082	0.8937	0.8952	0.9017	0.8020	0.9664
	MambaOnly	0.9090	0.9083	0.9096	0.9095	0.9089	0.8179	0.9780
	R-HMB-Net	0.9105	0.9098	0.9111	0.9110	0.9104	0.8209	0.9767
	P-HMB-Net	0.9161	0.9175	0.9146	0.9148	0.9161	0.8321	0.9806
	**HMB-Net**	**0.9264**	**0.9318**	**0.9210**	**0.9218**	**0.9268**	**0.8528**	**0.9844**

https://doi.org/10.3390/biology15212028
